# *O*-GlcNAcylation of Sox2 at threonine 258 regulates the self-renewal and early cell fate of embryonic stem cells

**DOI:** 10.1038/s12276-021-00707-7

**Published:** 2021-11-24

**Authors:** Dong Keon Kim, Jang-Seok Lee, Eun Young Lee, Hansol Jang, Suji Han, Hee Yeon Kim, In-Young Hwang, Ji-Woong Choi, Hyun Mu Shin, Hye Jin You, Hong-Duk Youn, Hyonchol Jang

**Affiliations:** 1 Anticancer Resistance Branch, Division of Rare and Refractory Cancer, Research Institute, National Cancer Center, Goyang, 10408 Republic of Korea; 2 National Creative Research Center for Epigenome Reprogramming Network, Department of Biomedical Sciences, Ischemic/Hypoxic Disease Institute, Seoul National University College of Medicine, Seoul, 03080 Republic of Korea; 3 Department of Molecular Medicine and Biopharmaceutical Sciences, Graduate School of Convergence Science and Technology, Seoul National University, Seoul, 03080 Republic of Korea; 4 Department of Cancer Biomedical Science, National Cancer Center Graduate School of Cancer Science and Policy, Goyang, 10408 Republic of Korea; 5 European Molecular Biology Laboratory, Genome Biology Unit, Heidelberg, Germany; 6 Wide River Institute of Immunology, Seoul National University, Hongcheon, 25159 Republic of Korea; 7 BK21 FOUR Biomedical Science Project & Department of Biomedical Sciences, Seoul National University College of Medicine, Seoul, 03080 Republic of Korea; 8 Cancer Microenvironment Branch, Division of Cancer Biology, Research Institute, National Cancer Center, Goyang, 10408 Republic of Korea

**Keywords:** Glycosylation, Embryonic stem cells

## Abstract

Sox2 is a core transcription factor in embryonic stem cells (ESCs), and *O*-GlcNAcylation is a type of post-translational modification of nuclear-cytoplasmic proteins. Although both factors play important roles in the maintenance and differentiation of ESCs and the serine 248 (S248) and threonine 258 (T258) residues of Sox2 are modified by *O*-GlcNAcylation, the function of Sox2 *O*-GlcNAcylation is unclear. Here, we show that *O*-GlcNAcylation of Sox2 at T258 regulates mouse ESC self-renewal and early cell fate. ESCs in which wild-type Sox2 was replaced with the Sox2 T258A mutant exhibited reduced self-renewal, whereas ESCs with the Sox2 S248A point mutation did not. ESCs with the Sox2 T258A mutation heterologously introduced using the CRISPR/Cas9 system, designated E14-Sox2^TA/WT^, also exhibited reduced self-renewal. RNA sequencing analysis under self-renewal conditions showed that upregulated expression of early differentiation genes, rather than a downregulated expression of self-renewal genes, was responsible for the reduced self-renewal of E14-Sox2^TA/WT^ cells. There was a significant decrease in ectodermal tissue and a marked increase in cartilage tissue in E14-Sox2^TA/WT^-derived teratomas compared with normal E14 ESC-derived teratomas. RNA sequencing of teratomas revealed that genes related to brain development had generally downregulated expression in the E14-Sox2^TA/WT^-derived teratomas. Our findings using the Sox2 T258A mutant suggest that Sox2 T258 *O*-GlcNAc has a positive effect on ESC self-renewal and plays an important role in the proper development of ectodermal lineage cells. Overall, our study directly links *O*-GlcNAcylation and early cell fate decisions.

## Stem cell development: hold the sugar

Cells that can grow into any type of cell, called embryonic stem cells (ESCs), are signaled to stay in stem cell mode (maintain stemness) by addition of a single sugar molecule to Sox2, a regulatory protein coded for by a developmental gene. Sugar modification of Sox2 was known to be involved in maintaining stemness and sometimes implicated in cancer, but the mechanism was poorly understood. When Hyonchol Jang at the National Cancer Center in South Korea and co-workers prevented sugar modification of Sox2 by changing an amino acid at the sugar-binding site, ESCs showed reduced self-renewal. Rather than repressing genes related to stemness, the modification failed to repress developmental genes, permitting cells to grow into other cell types. These results illuminate both the role of Sox2 in cancer and the importance of sugar modification in stemness.

## Introduction

*O*-GlcNAcylation, which entails attachment of a single monosaccharide, *N*-acetyl-d-glucosamine (GlcNAc), to a serine or threonine residue of a nucleocytoplasmic protein via an *O*-β-glycosidic linkage, is associated with the sensing of nutrients^1,2^ and affects embryonic stem cell (ESC) pluripotency^3–5^. In *O*-GlcNAcylation, GlcNAc is attached and removed by only one enzyme in each process, *O*-GlcNAc transferase (Ogt) and *O*-GlcNAcase (Oga), respectively^1,2^. Decreased expression of Ogt leads to downregulation of the global *O*-GlcNAc level, which in turn was shown to reduce mouse ESC self-renewal and the efficiency of reprogramming mouse embryonic fibroblasts (MEFs) to induce pluripotent stem cells (iPSCs)^3^. Chemical inhibition of Ogt decreased global *O*-GlcNAc levels and accelerated the differentiation of human ESCs^6^. Upregulation of global *O*-GlcNAc levels, either via enhanced Ogt expression or glucose levels in the culture medium, increased the efficiency of reprogramming MEFs into iPSCs^3^. Similarly, upregulation of global *O*-GlcNAc levels via chemical inhibition of Oga suppressed mouse ESC differentiation^7^. The key pluripotent transcription factors Oct4 and Esrrb are modified by *O*-GlcNAc at the threonine 228 (T228) and serine 25 (S25) residues, respectively, with *O*-GlcNAcylation at these residues enhancing ESC self-renewal^3,5^.

The SRY family transcription factor Sox2, together with Oct4 and Nanog, constitutes the core circuit in the pluripotency gene regulatory network^8,9^. Under self-renewal conditions, Sox2 not only regulates the expression of pluripotency genes but also suppresses the expression of lineage-differentiation genes, thus maintaining ESC pluripotency^10,11^. Sox2-null embryos died shortly after implantation due to failed pluripotent epiblast formation^12^, demonstrating the importance of Sox2 in ESC maintenance. During in vitro ESC differentiation, Sox2 expression is downregulated but still maintained in the neural ectoderm, where it promotes neural ectodermal differentiation while inhibiting mesendodermal differentiation^13^. Similarly, Sox2 induction resulted in a neural plate fate for differentiating axial stem cells in vivo^14^. These findings show that Sox2 plays an important role in determining early cell fate as well as in maintaining the pluripotency of ESCs^15,16^.

In addition to ESCs, Sox2 is expressed in various adult stem and progenitor cells, including those in the lung, stomach, brain, skin, and bone, and can regulate their properties^15^. Sox2 is also closely associated with developmental disorders and multiple cancers, including lung squamous cell carcinoma, glioma, and melanoma^15,17^. Due to the importance of Sox2 in these various physiological phenomena, many studies have evaluated the molecular function and regulation of Sox2. Sox2 undergoes numerous types of post-translational modifications (PTMs), such as phosphorylation, acetylation, methylation, SUMOylation, ubiquitination, PARylation, and *O*-GlcNAcylation^15,16,18^. Although some PTMs have been found to influence the transcriptional activity of Sox2 by controlling its stability, nuclear–cytoplasmic localization, or transactivation potential^15^, the functions of many PTMs have not yet been identified.

*O*-GlcNAcylation at the positions corresponding to amino acids 248–264 of murine Sox2 was first identified via liquid chromatography–tandem mass spectrometry analysis of the rat brain^19^. When all nine serine and threonine sites of this sequence were point-mutated to alanine, succinylated wheat germ agglutinin (sWGA) bead binding assays indicated that S248, T258, and S259 are potential *O*-GlcNAcylation sites in mouse ESCs^3^. High-resolution electron transfer dissociation tandem mass spectrometry analysis of mouse ESC nuclear proteins confirmed that S248 and T258 can be modified by *O*-GlcNAcylation^20^. Double Sox2 mutations at T258 and S259 to alanine (T258A/S259A) reduced Sox2’s ability to reprogram MEFs into iPSCs, whereas a triple mutation (S248A/T258A/S259A) did not^3^. Sox2 S248A also increased the somatic cell reprogramming efficiency of Sox2^21^. These results imply that Sox2 may have different functions depending on the residues affected by *O*-GlcNAc modification.

In the present study, we investigated the role of Sox2 *O-*GlcNAcylation in the maintenance of mouse ESC self-renewal and in early differentiation in vivo. Sox2 T258A reduced ESC self-renewal in conventional in vitro culture medium containing serum and leukemia inhibitory factor (LIF), whereas S248A did not. During differentiation via teratoma formation in mouse xenografts, Sox2 T258A resulted in biased lineage differentiation. RNA sequencing (RNA-seq) analysis indicated that Sox2 T258A cannot adequately repress developmental genes. These findings help deepen our understanding of the role of *O*-GlcNAcylation in ESCs and serve as a basis for further investigation of the roles of Sox2 *O*-GlcNAcylation in cancer and other diseases.

## Materials and methods

### Cell culture and chemicals

E14 and 2TS22C mouse ESCs, which have been described previously, were cultured in the presence of serum and LIF^3,22^. NIH3T3 cells were cultured as described previously^3^. Cell lines were authenticated using short tandem repeat profiling and were regularly examined for *Mycoplasma* at the Genomics Core Facility (National Cancer Center, Korea) as described previously^23^. *N*-acetyl glucosamine (#A4106), doxycycline (Dox; #9891), blasticidin (#15205), and CHIR99021 (#SML1046) were purchased from Sigma-Aldrich (USA). PD0325901 was purchased from Selleck Chemicals (#S1036; USA).

### sWGA pulldown and Western blot analysis

Agarose-bound sWGA was purchased from Vector Laboratories (#AL-1023S; USA). The sWGA pulldown assay was performed according to the manufacturer’s instructions. *O*-GlcNAcylated proteins were eluted with elution buffer containing 0.1 M *N*-acetyl glucosamine at room temperature for 20 min. Western blot analysis was performed according to a modified version of a previously described method^24^. Cells were lysed with lysis buffer containing a protease inhibitor cocktail (P3100; genDEPOT, USA) and a phosphatase inhibitor cocktail (P3200; genDEPOT). After sodium dodecyl sulfate-polyacrylamide gel electrophoresis and protein transfer, the target protein was detected using the iBind™ Automated Western system (Thermo Fisher Scientific, USA). Anti-FLAG (#F1804) and anti-ActB (#AC-74) antibodies were purchased from Sigma-Aldrich. Anti-Sox2 (ab97959) antibody was purchased from Abcam (UK), and anti-Oct4 (sc-5279) antibody was purchased from Santa Cruz Biotechnology (USA).

### Retroviral expression of Sox2 mutants and self-renewal assay

For retroviral expression of Sox2, murine wild-type (WT) Sox2 and mutant Sox2 (S248A, S248D, T258A) were subcloned into the pMSCV-FLAG puro vector^3^. Retroviruses were produced using the Plat-E system as described previously^3^. Next, 2TS22C cells were infected with retroviruses in the presence of 8 µg/ml polybrene (#H9268, Sigma). At 24 h after infection, Dox (1 μg/ml) was added to eliminate endogenous Sox2, and approximately 7 days later, ESC colonies were stained with alkaline phosphatase (AP) as described previously^25^.

### Expression of CAG promoter-driven FLAG-Sox2 mutants

For long-term transgene expression in ESCs, WT and mutant Sox2 (S248A, T258A, S248A/T258A) were subcloned into pCAG-FLAG-IRES-blasticidin vectors^26^. Then, 2TS22C cells were transfected with Lipofectamine 2000 (Invitrogen, USA). At 2 days after transfection, blasticidin (15 μg/ml) and Dox (1 μg/ml) was added to the cells for at least 14 days. After selection, the cells were maintained in the presence of Dox (1 μg/ml).

### CRISPR/Cas9-mediated alanine mutation of Sox2 T258

Alanine mutation of Sox2 T258 in E14 cells was performed as described previously^27^ with some modifications. A guide RNA sequence near T258 (TTACCTCTTCCTCCCACTCC) was inserted into the PX458 vector (#48138; Addgene, USA), which was then named PX458-gSox2^T258^. As a donor, single-stranded DNA (120 bp, CTCCATGGGCTCTGTGGTCAAGTCCGAGGCCAGCTCCAGC CCCCCCGTGGTTGCCTCTTCCTCCCACTCCAGAGCGCCCTGCCAGGCCGGGGACCTCCGGGACATGATCAGCATGTACCT) was synthesized at Macrogen (Seoul, Korea). At 3 days after transfection of PX458-gSox2^T258^ and donor single-stranded DNA into E14 cells using Lipofectamine 2000, GFP-positive cells were sorted using a FACSMelody sorter (BD Biosciences, USA) at the Flow Cytometry Core Facility (National Cancer Center). Individual cells were left to proliferate in a 96-well culture plate in the presence of 2i (PD0325901 and CHIR99021)^13^, and clones with alanine mutations were selected using genomic DNA sequencing. The selected clones were normally maintained in serum with LIF and 2i. Cells at passages 1–10 after removal of 2i were used for the experiments. The following primers were used to confirm the presence of the T258A mutant: forward, 5′-CCTACATGAACGGCTCGCCC-3′; reverse, 5′-CTCCTCTTTTTGCACCCCTCC-3′.

### Immunofluorescence staining and confocal microscopy

Immunofluorescence staining was performed as described previously^28^. Briefly, ESCs were fixed with 4% (w/v) paraformaldehyde and permeabilized with 0.5% (w/v) Triton X-100 for 10 min. After blocking with 2% bovine serum albumin in phosphate-buffered saline (PBS), the ESCs were stained with an anti-Sox2 antibody and incubated overnight at 4 °C. After 24 h, the samples were incubated with Alexa Fluor 488-conjugated anti-mouse antibody (Invitrogen) for 30 min at room temperature. Images were obtained under 20× magnification at the Image Core of the National Cancer Center using the LSM510 META microscope (Carl Zeiss, Germany).

### Real-time imaging of ESCs

Real-time imaging of ESCs was achieved using the Operetta CLS High-Content Analysis System (PerkinElmer, USA). Briefly, 1 × 10^3^ cells were seeded onto a CellCarrier-96 Ultra microplate (#6055300; PerkinElmer) precoated with 0.1% gelatin. After 6 h, the plate was loaded onto the Operetta system, and images were taken at 1-h intervals for 60 h under 20× magnification. The results were analyzed using Harmony High Content Analysis software (PerkinElmer).

### RNA-seq and bioinformatics analysis

Total RNA extraction, preparation of an RNA library, and RNA-seq and bioinformatics analyses were performed as described previously^29,30^ with some modifications. RNA-seq was performed using the Nova-Seq 6000 sequencing system (Illumina, USA) by Macrogen. The RNA-seq data were deposited in the Gene Expression Omnibus database under the accession numbers GSE176211 and GSE176212. Gene set enrichment analysis (GSEA) was performed using GSEA version 4.1.0 software. A self-renewal gene set and early differentiation gene set were extracted from GSE36322^3^. Genes with at least twofold upregulation in expression in E14 cells before differentiation compared with E14 cells at 2 days after differentiation into embryoid bodies were considered to be self-renewal genes, whereas genes with downregulated expression at least twofold were considered differentiation genes.

Differentially expressed genes (DEGs) were filtered based on a fold change > 2. Genes with transcripts per million values <10 in all samples were excluded from the subsequent analysis. DEGs were subjected to core analysis using Ingenuity Pathway Analysis (IPA) software (Qiagen, USA). In particular, the DEGs were subjected to subanalyses of diseases and functions, canonical pathways, and upstream regulators.

Sox2 chromatin immunoprecipitation-sequencing (ChIP-seq) data were processed as described previously^31^ using GSE44288^32^. Sox2 occupancy at putative target gene loci in the ESCs was visualized using the Integrated Genomics Viewer^33^.

### Animal experiments

Animal experiments were performed as described previously^25^, with some modifications. E14 or E14-Sox2^TA/WT^ cells were dissociated and suspended in PBS supplemented with 50% Matrigel (#354234; BD Biosciences, USA) at a density of 1 × 10^7^ cells/ml. ESCs (100 μl) were inoculated subcutaneously into each BALB/c-nu mouse (Orient Bio, Korea). Three mice were inoculated per group. At 14 days after inoculation, the mice were sacrificed, and teratomas were harvested for further analysis. This study was reviewed and approved by the Institutional Animal Care and Use Committee of the National Cancer Center Research Institute (NCC-19-489).

### Histological analysis of teratomas

Teratomas were dissected and fixed in 4% paraformaldehyde overnight. After microdissection, sections were stained with hematoxylin and eosin (H&E) as described previously^25^ at the Laboratory Animal Research Facility (National Cancer Center). H&E-stained slides were imaged at high resolution (200×) using the Phenochart 1.0.12 viewer from the Vectra Polaris 1.0 imaging system (PerkinElmer). Measurements were obtained from the image files using InForm 2.4.10 image analysis software (PerkinElmer). The H&E-stained slides were analyzed by a pathologist.

### Reverse-transcription PCR and real-time quantitative PCR

Reverse-transcription PCR and real-time quantitative PCR (qPCR) were performed according to a previously described method^23^. The primers were as follows: ActB (forward (fwd): 5′-ATCACTATTGGCAACGAGCG-3′, reverse (rev): 5′- TCAGCAATGCCTGGGTACAT-3′); 18S rRNA (fwd: 5′-TTAGAGTGTTCAAAGCAGGCCCGA-3′, rev: 5′- TCTTGGCAAATGCTTTCGCTCTGG-3′); Cdx2 (fwd: 5′-CTCCGAGAGGCAGGTTAAAA-3′, rev: 5′-AGGAGGTCACAGGACTCAAG-3′); T (fwd: 5′-GTGAAGGTGGCTGTTGGGTA-3′, rev: 5′-CACTCGCAGTTCGCGTTC-3′); Twist2 (fwd: 5′-CTTCCTCTACCAGGTTCTCC-3′, rev: 5′-AGGTGGGTCCTGGCTT -3′); Eomes (fwd: 5′-TCTGCACAAATACCAACCGA-3′, rev: 5′-AGCCGTGTACATGGAATCGTA-3′); Afp (fwd: 5′-GGGAATGGCCGACATTTTCAT-3′, rev: 5′-AGCTTGGCACAGATCCTTGT-3′); Foxa2 (fwd: 5′-GCCCGAGGGCTACTCTTC-3′, rev: 5′-ATTCCAGCGCCCACATAGG-3′); Krt8 (fwd: 5′-CGGCTACTCAGGAGGACTGA-3′, rev: 5′-CAGCTTCCCATCTCGGGTTT-3′); Sox17 (fwd: 5′-CAGGGTCTGGCCTGAATGTT-3′, rev: 5′-AAAGGCGCAGTCTCTTCTCC-3′); Sox1 (fwd: 5′-GCAGCGTTTCCGTGACTTTAT-3′, rev: 5′-GGCAGAACCACAGGAAAGAAA-3′); Tubb3 (fwd: 5′-GGCAACTATGTAGGGGACTC-3′, rev: 5′-GCACCACTCTGACCAAAGAT-3′); Nestin (fwd: 5′-GGAAGTGGCTACATACAGGACT-3′, rev: 5′-GGGTATTAGGCAAGGGGGAAG-3′); Pax6 (fwd: 5′-TGTCAGATCTGCTACTTCCCC-3′, rev: 5′-CTCGAATACGGGGCTCTGA-3′); Otx2 (fwd: 5′-AGCAAATCTCCCTGAGAGCG-3′, rev: 5′-AGTGACGGAACTTACAGCCG-3′); Tbx3 (fwd: 5′-CCACCCGTTCCTCAATTTGAACAG-3′, rev: 5′-CGGAAGCCATTGATGGTAAAGCTG -3′); Mylpf (fwd: 5′-GCCCCCAGGAGATCTAAGAC-3′, rev: 5′-CCACTGGCTTCCTTCATCAT-3′); Pou3f1 (fwd: 5′-TCGAGGTGGGTGTCAAAGG-3′, rev: 5′-GGCGCATAAACGTCGTCCA-3′); and Krt18 (fwd: 5′-AGATGACACCAACATCACAAGG -3′, rev: 5′-TCCAGACCTTGGACTTCCTC-3′).

### Statistical analysis

Statistical analysis was performed as reported previously^34^. Numerical values are expressed as the mean ± standard deviation. The significance of differences between two groups was evaluated using a two-tailed, unpaired Student’s *t* test.

## Results

### Sox2 T258 *O*-GlcNAcylation-defective mutation reduced ESC maintenance

**Fig. 1:**
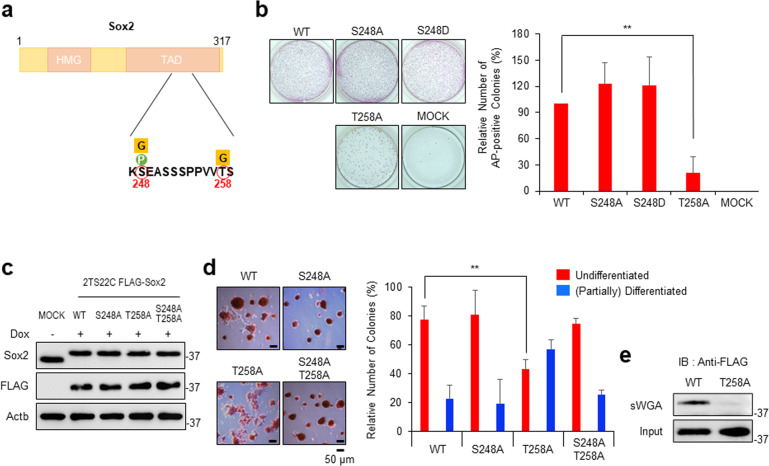
An *O-*GlcNAcylation-defective mutation at Sox2 threonine 258 (T258) reduces embryonic stem cell (ESC) maintenance. **a** A schematic diagram of the Sox2 protein domain^9^ and *O*-GlcNAc-modified residues previously revealed by mass spectrometry^20^ is shown. HMG high-mobility group, TAD transactivation domain, G *O*-GlcNAc-modified residues, P phosphorylated residue. **b** Equal numbers of 2TS22C cells were infected with the same titers of empty retroviruses (MOCK) or retroviruses containing wild-type (WT) Sox2 or Sox2 point mutants (S248A, S248D, T258A). After elimination of endogenous Sox2 by treatment with doxycycline (Dox) for 7 days, ESC colonies were stained with alkaline phosphatase (AP). Representative images (left) and the proportions of AP-positive colonies (right) are shown. Values are expressed as percentages (mean ± standard deviation, *n* = 3). **P < 0.01. **c** CAG promoter-driven FLAG-Sox2 mutants (WT, S248A, T258A, S248A/T258A) were introduced into 2TS22C cells in the presence of Dox. After the selection of cells stably expressing FLAG-Sox2, the expression of endogenous and exogenous Sox2 was evaluated using Western blot analysis. As a control, an empty vector was introduced into 2TS22C cells in the absence of Dox. **d** The cells described in **c** were replated for a self-renewal assay. The undifferentiated state was assessed based on both morphology and AP staining. Relative colony numbers are expressed as percentages (mean ± standard deviation, *n* = 3). **P < 0.01. **e** Whole-cell lysates from 2TS22C cells expressing WT FLAG-Sox2 or FLAG-Sox2 T258A as described in (**c**) were pulled down using succinylated wheat germ agglutinin (sWGA) beads and immunoblotted with an anti-FLAG antibody.

*O-*GlcNAc modifications at the S248 and T258 residues within the transactivation domain of Sox2^3,20,21^, as well as phosphorylation at S248^35,36^, have been reported previously (Fig. 1a). To determine the role of each PTM in ESCs, we generated retroviruses harboring WT Sox2, Sox2 alanine mutants (S248A, T258A), and a Sox2 aspartate mutant (S248D). PTMs cannot occur in alanine mutants, whereas an aspartate mutant mimics phosphorylation. Then, 2TS22C ESCs, in which Sox2 protein expression was completely depleted within 2 days after the addition of Dox^37^, were infected with retroviruses and treated with Dox at 24 h after infection. In the self-renewal assay, the cells infected with retroviruses containing WT Sox2 produced many AP-positive colonies, whereas the cells infected with empty retroviruses rarely produced AP-positive colonies (Fig. 1b). The cells infected with Sox2 T258A produced significantly fewer AP-positive colonies than those infected with WT Sox2, whereas the cells infected with Sox2 S248A and S248D did not (Fig. 1b). In NIH3T3 cells infected with the same amount of retrovirus, WT and mutant Sox2 proteins were expressed at almost equal levels (Supplementary Fig. 1), indicating that the previous result was not caused by a difference in the viral titers. Because retroviral promoters are commonly silenced during long-term ESC culture^38^, gene expression using retroviruses is inadequate for observing mid- to long-term effects of Sox2 mutation. Thus, 2TS22C cells stably expressing WT and mutant FLAG-Sox2 under the control of the constitutive CAG expression unit were generated^39^. Western blot analysis showed that both WT and mutant FLAG-Sox2 were expressed at similar levels, and the amount of exogenously expressed Sox2 in the Dox-treated cells was close to the original endogenous Sox2 level (Fig. 1c). The self-renewal assay using these cells showed that alanine mutation at T258 significantly reduced the number of undifferentiated ESC colonies (Fig. 1d). Simultaneous alanine mutation at S248 and T258 did not significantly affect the number of undifferentiated ESC colonies (Fig. 1d), suggesting that PTM at S248 may negatively affect the self-renewal of the cells. A pulldown assay of *O*-GlcNAc-modified proteins using sWGA beads in the 2TS22C FLAG-Sox2 WT and FLAG-Sox2 T258A cell lysates followed by immunoblotting with an anti-FLAG antibody showed an apparent reduction in the level of *O*-GlcNAc modification at T258A (Fig. 1e). These results suggest that *O*-GlcNAcylation at T258 is important for ESC maintenance.

### CRISPR/Cas9-mediated alanine mutation of Sox2 T258 in one allele reduced ESC self-renewal

**Fig. 2:**
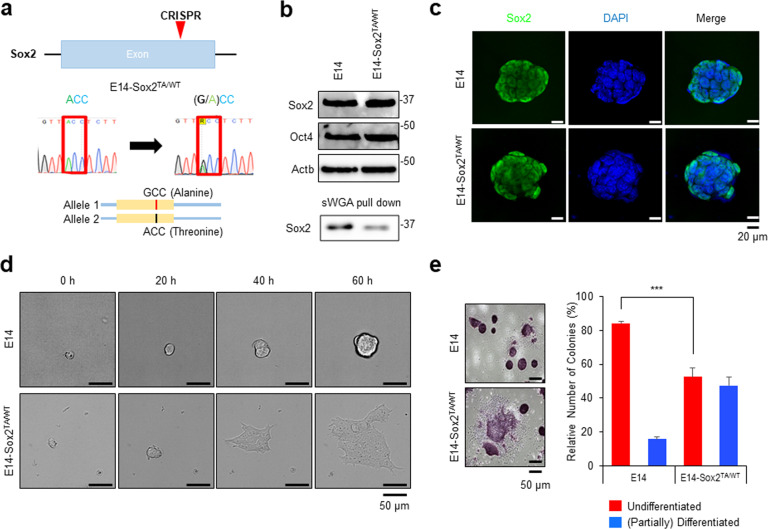
CRISPR/Cas9-mediated alanine mutation of Sox2 T258 in one allele reduces ESC maintenance. **a** A schematic diagram of the threonine-to-alanine mutation at Sox2 T258 is shown. In E14 cells, Sox2 was mutated using a CRISPR/Cas9-mediated knock-in system. Colonies containing the Sox2 T258 mutation were selected after colony expansion from single cells. Genomic DNA sequencing showed that the Sox2 T258 mutation was introduced into one allele, with the other allele remaining wild type; thus, the cells were named E14-Sox2^TA/WT^. **b** Sox2 and Oct4 protein expression levels were evaluated using Western blot analysis (top). Whole-cell lysates from the E14 and E14-Sox2^TA/WT^ cells were pulled down using sWGA beads and immunoblotted with an anti-Sox2 antibody (bottom). **c** Cellular localization of Sox2 in the E14 and E14-Sox2^TA/WT^ cells was observed by immunofluorescence confocal microscopy using an LSM510 META microscope (Carl Zeiss, Germany). **d** Time-course images of the E14 and E14-Sox2^TA/WT^ cells under self-renewal conditions. Bright-field images were acquired using the Operetta system (PerkinElmer) at 1-h intervals for 60 h. Representative images are shown, and full movies can be found in Supplementary Movies 1 and 2. **e** The E14 and E14-Sox2^TA/WT^ cells were replated and subjected to a self-renewal assay. AP staining was performed 5 days after replating. Relative colony numbers are expressed as percentages (mean ± standard deviation, *n* = 3). ***P < 0.001.

Because Sox2 is precisely regulated at various levels from transcription to PTM, the use of systems in which Sox2 is expressed by foreign promoters may not represent actual physiological conditions. To overcome these limitations, we introduced the Sox2 T258A mutation into an endogenous chromosome in E14 cells using the CRISPR/Cas9 gene-editing system. Despite several efforts, E14 cells harboring the Sox2 T258A mutation in both alleles were not found. Therefore, E14 cells harboring the Sox2 T258A mutation in one allele, with the other allele encoding WT Sox2, were selected and named E14-Sox2^TA/WT^ cells (Fig. 2a).

Western blot and sWGA bead pulldown analyses showed that the Sox2-*O*-GlcNAcylation levels were decreased in the E14-Sox2^TA/WT^ cells, whereas the protein expression levels of Sox2 and Oct4 were similar in both cell lines (Fig. 2b). The cycloheximide chase assay showed no significant differences in Sox2 protein stability between the E14 and E14-Sox2^TA/WT^ cells (Supplementary Fig. 2). Intracellularly, Sox2 was predominantly located in the nucleus in both cell types, as revealed by confocal microscopy after immunofluorescence staining (Fig. 2c). Three-dimensional z-stack images of the 2TS22C FLAG-Sox2 WT and FLAG-Sox2 T258A cells showed that both Sox2 WT and Sox2 T258A were predominantly present in the nucleus without any particular differences in their subcellular location (Supplementary Fig. 3). These results suggest that Sox2 T258 *O*-GlcNAcylation does not affect Sox2 protein stabilization and subcellular localization.

Although only one allele harbored the mutation, the E14-Sox2^TA/WT^ cells exhibited reduced self-renewal. Time-lapse images of the E14 and E14-Sox2^TA/WT^ cells under self-renewal conditions showed that the E14 cells developed a dome shape as they proliferated, whereas the E14-Sox2^TA/WT^ cells flattened out during proliferation (Fig. 2d, Supplementary Movie 1, and Supplementary Movie 2). The self-renewal assay showed that approximately half of the E14-Sox2^TA/WT^ cells developed a dome shape as they grew and were strongly AP-positive, whereas the other half flattened out as they grew and were weakly AP-positive (Fig. 2e). These results imply that *O*-GlcNAcylation at Sox2 T258 is required for the efficient self-renewal of ESCs.

### Genes influenced by *O*-GlcNAcylation at Sox2 T258 under self-renewal conditions

**Fig. 3:**
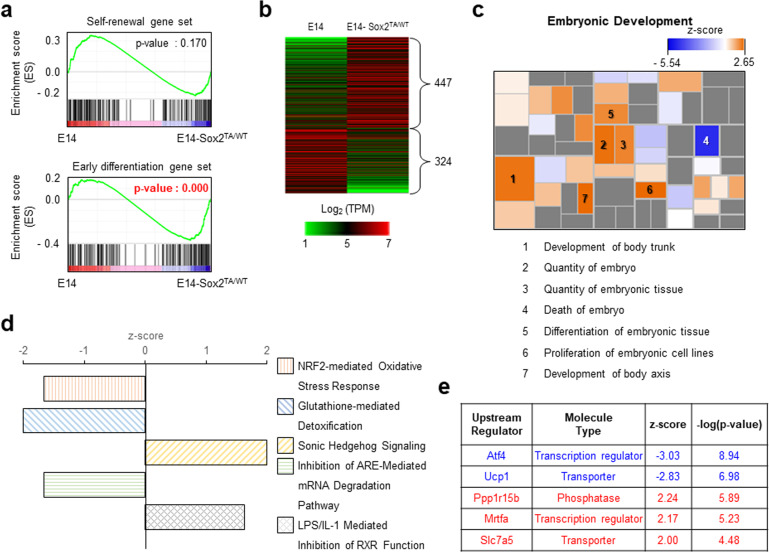
RNA sequencing (RNA-seq) showed enrichment of development-related genes in the E14-Sox2^TA/WT^ cells under self-renewal conditions. **a** Whole transcriptomes of the E14 and E14-Sox2^TA/WT^ cells were analyzed by RNA-seq. Gene set enrichment analysis revealed enrichment of early differentiation genes, but not self-renewal genes, in the E14-Sox2^TA/WT^ cells. **b** Differentially expressed genes (DEGs) with a greater than twofold change in expression are represented in a heat map. The heat map was generated using MeV 4.9. TPM transcripts per million. **c** DEGs were investigated using Ingenuity Pathway Analysis (IPA). Analyses of diseases and functions showed that embryonic development-related pathways were generally activated in the E14-Sox2^TA/WT^ cells. Pathways with an absolute *z*-score ≥ 1 are presented in −log(*p* value) order. **d** The most significantly affected signaling pathways were examined by canonical pathway analysis in IPA. Pathways with an absolute *z*-score ≥ 1.5 are listed in −log(*p* value) order. The top five pathways are shown. **e** Putative upstream regulators were investigated using IPA. Regulators with an absolute *z*-score ≥ 2 are listed in −log(*p* value) order. The top five regulators are listed.

To investigate the molecular changes resulting from changes in the degree of *O*-GlcNAcylation at Sox2 T258, we analyzed the whole transcriptomes of the E14 and E14-Sox2^TA/WT^ cells using RNA-seq. GSEA showed that the self-renewal gene set did not differ between the E14 and E14-Sox2^TA/WT^ cells, whereas the early differentiation gene set was significantly different (Fig. 3a). Of the DEGs exhibiting a greater than twofold change, 447 genes had upregulated expression and 324 genes had downregulated expression in the E14-Sox2^TA/WT^ cells compared with the E14 cells (Fig. 3b). IPA of the diseases and functions of the DEGs revealed that in the E14-Sox2^TA/WT^ cells, embryonic development-related pathways were generally activated, whereas an embryo death-related pathway was suppressed (Fig. 3c). Canonical pathway analysis of DEGs using IPA revealed that the most significantly altered pathways were the nuclear factor erythroid 2-like 2 (Nrf2)-mediated oxidative stress response, glutathione-mediated detoxification, sonic-hedgehog signaling, inhibition of the AU-rich element (ARE)-mediated mRNA degradation pathway, and lipopolysaccharide/interleukin-1-mediated inhibition of RXR function (Fig. 3d). Consistent with the phenotypes, Nrf2^40^, glutathione peroxidase-1^41^, and the ARE-mediated mRNA degradation pathway^42^ were shown to be involved in the regulation of ESC self-renewal. Upstream regulator analysis showed that Atf4 and Ucp1 function was inhibited, whereas Ppp1r15b, Mrtfa, and Slc7a5 function was activated (Fig. 3e). Among the identified regulators, Atf4 positively regulates ESC self-renewal^43^.

**Fig. 4:**
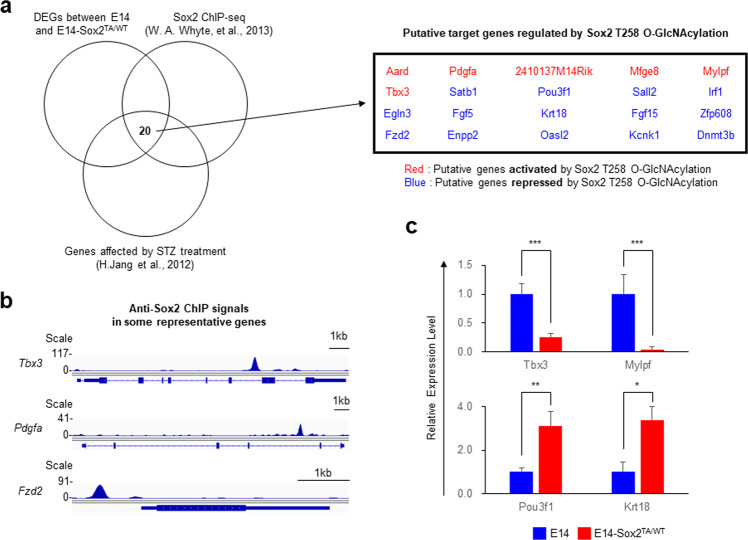
Identification of genes regulated by Sox2 T258 *O*-GlcNAcylation. **a** A schematic diagram and list of the putative genes regulated directly by Sox2 T258 *O*-GlcNAcylation are shown. Genes that produced anti-Sox2 antibody ChIP signals in the gene region (GSE44288)^32^, those with downregulated expression in the E14-Sox2^TA/WT^ cells, and those with upregulated expression after streptozotocin (STZ) treatment (GSE36322)^3^ were considered putative genes activated by Sox2 T258 *O*-GlcNAcylation. Repressed genes were also defined based on similar logic. **b** Anti-Sox2 antibody ChIP-seq binding profiles are shown for certain putative genes regulated by Sox2 T258 *O*-GlcNAcylation. Sox2 occupancy at putative target gene loci in ESCs was visualized using Integrated Genomics Viewer^33^. **c** The expression of some putative target genes was examined in the E14 and E14-Sox2^TA/WT^ cells using qPCR. The graph presents the relative expression levels (mean ± standard deviation, *n* = 3) after normalization to 18*S* rRNA expression. *P < 0.05, **P < 0.01, ***P < 0.001.

To identify target genes directly affected by *O*-GlcNAcylation at T258, we performed a combination of RNA-seq, ChIP-seq, and microarray analyses. DEGs identified by RNA-seq in the E14 and E14-Sox2^TA/WT^ cells represent putative genes regulated by inhibition of Sox2 T258 *O*-GlcNAcylation. Genes with anti-Sox2 antibody ChIP signals in the gene region (GSE44288)^31,32^ represent putative genes directly regulated by Sox2. DEGs identified by microarray analysis in the E14 cells treated with or without streptozotocin (STZ) upon differentiation into embryoid bodies (GSE36322)^3^ represent putative genes regulated by *O*-GlcNAcylation. STZ elevates the global level of *O*-GlcNAc by inhibiting Oga^3^. Six genes (*Aard, Pdgfa, 2410137M14Rik, Mfge8, Mylpf*, and *Tbx3*) were identified as putative genes activated directly by Sox2 T258 *O*-GlcNAcylation. These genes had a Sox2 ChIP signal in their gene region and showed downregulated expression in the E14-Sox2^TA/WT^ cells and upregulated expression after STZ treatment. Eighteen putative genes (*Satb1, Pou3f1, Sall2, Irf1, Egln3, Bcl11b, Fgf5, Cxcl12, Krt18, Pcsk9, Fgf15, Rab25, Zfp608, Fzd2, Enpp2, Oasl2, Kcnk1*, and *Dnmt3b*) that may be directly repressed by Sox2 T258 *O*-GlcNAcylation were identified based on similar logic (Fig. 4a, b). Tbx3 plays an essential role in the maintenance of self-renewal^44^, and Satb1-null ESCs were shown to exhibit impaired differentiation^45^. For some putative direct target genes, the RNA-seq results were validated by real-time qPCR (Fig. 4c). These findings indicate that Sox2 T258 *O*-GlcNAcylation influences target gene selection and that the decreased self-renewal ability of E14-Sox2^TA/WT^ cells is primarily due to the expression of differentiation-related genes rather than a decrease in the expression of self-renewal genes.

### Teratomas derived from E14-Sox2^TA/WT^ exhibited a propensity for lineage bias

**Fig. 5:**
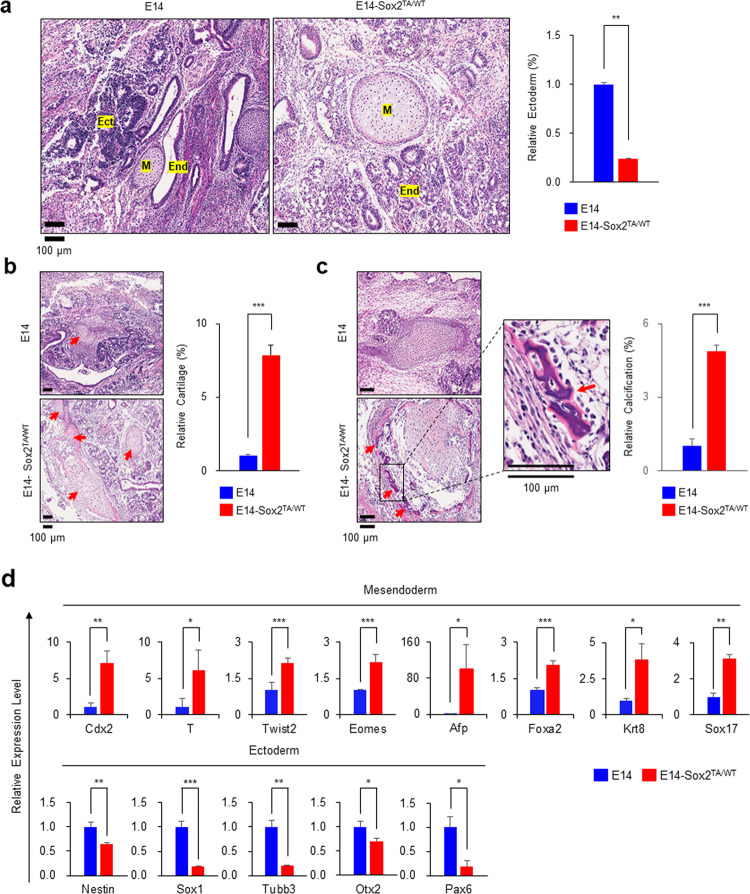
Teratomas derived from the E14-Sox2^TA/WT^ cells exhibited decreased ectodermal lineage commitment and increased cartilage formation. **a** The E14 and E14-Sox2^TA/WT^ cells were transplanted into nude mice. Teratomas that formed 14 days after transplantation were analyzed by hematoxylin and eosin staining. The E14-Sox2^TA/WT^ cells exhibited decreased ectodermal lineage commitment. Scale bars, 100 μm. Ect ectodermal lineage, M mesodermal lineage, End endodermal lineage. The degree of ectodermal lineage commitment was quantified using InForm 2.4.10 image analysis software (PerkinElmer), and the relative amounts of ectoderm formed (%) are presented as the mean ± standard deviation (*n* = 3). **b** The E14-Sox2^TA/WT^ cells exhibited increased cartilage formation. Representative images and the quantification results are shown. Red arrows indicate cartilage. **c** The E14-Sox2^TA/WT^ cells exhibited increased calcification. Representative images and the quantification results are shown. Red arrows indicate calcified tissue. **d** The mRNA expression levels of mesendodermal and ectodermal markers in teratomas were analyzed using real-time qPCR. The graph shows the relative expression levels (mean ± standard deviation, *n* = 3) after normalization to *ActB* expression. *P < 0.05, **P < 0.01, ***P < 0.001.

To investigate the effect of Sox2 T258 *O*-GlcNAcylation on ESC differentiation, we performed teratoma formation assays. Three nude mice per group were injected subcutaneously with E14 or E14-Sox2^TA/WT^ cells and then sacrificed 14 days later for teratoma analysis. Both E14 and E14-Sox2^TA/WT^ cells produced teratomas in all mice. Pathological analysis after H&E staining of paraformaldehyde-fixed microdissections showed that the E14-Sox2^TA/WT^ cells resulted in significantly lower numbers of ectoderm-lineage cells than the E14 cells (Fig. 5a and Supplementary Fig. 4). Another pathological feature was a marked increase in cartilage tissue of mesodermal origin from the E14-Sox2^TA/WT^ cells (Fig. 5b). The degree of calcification of the cartilage tissue also increased (Fig. 5c), indicating an increase in the maturity of the cartilage tissue. Real-time qPCR analysis of marker genes for mesendoderm (*Cdx2*, *T*, *Twist2*, *Eomes*, *Afp*, *Foxa2*, *Krt8*, and *Sox17*) and ectoderm (*Nestin*, *Sox1*, *Tubb3*, *Otx2*, and *Pax6*) in frozen teratomas derived from the E14 and E14-Sox2^TA/WT^ cells showed significantly upregulated expression of mesendodermal genes and downregulated expression of ectodermal genes in the E14-Sox2^TA/WT^ cells (Fig. 5d).

**Fig. 6:**
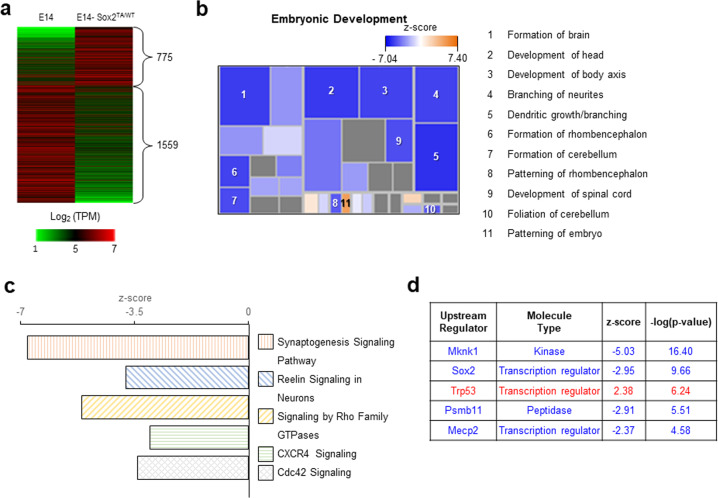
The teratomas derived from the E14-Sox2^TA/WT^ cells exhibited decreased neural differentiation according to RNA-seq. **a** DEGs in teratomas derived from the E14 versus E14-Sox2^TA/WT^ cells were analyzed using RNA-seq. DEGs with a greater than twofold change in expression are represented in a heat map. **b** DEGs were investigated using IPA. The analysis of diseases and functions showed that brain development-related pathways were generally suppressed in the E14-Sox2^TA/WT^ cells. Pathways with an absolute *z*-score of 2 or more are shown in −log(*p* value) order. **c** The most significantly affected signaling pathways were investigated by canonical pathway analysis in IPA. Pathways with an absolute *z*-score ≥ 2 are listed in −log(*p* value) order. The top five pathways are listed. **d** Putative upstream regulators were examined using IPA. Regulators with an absolute *z*-score ≥ 2 are listed in −log(*p* value) order. The top five regulators are listed.

To investigate the molecular changes, we analyzed whole transcriptomes of teratomas derived from the E14 and E14-Sox2^TA/WT^ cells using RNA-seq. Of the DEGs exhibiting a greater than twofold change, 775 showed upregulated expression and 1559 showed downregulated expression in the E14-Sox2^TA/WT^ teratomas compared with the E14 teratomas (Fig. 6a). IPA of the diseases and functions associated with the DEGs showed that brain development-related pathways were generally suppressed in the E14-Sox2^TA/WT^ teratomas (Fig. 6b), implying poor development of the ectodermal lineage. Canonical pathway analysis of DEGs using IPA revealed that the most significantly altered pathways were the synaptogenesis signaling pathway, reelin signaling in neurons, signaling involving Rho family GTPases, CXCR4 signaling, and Cdc42 signaling, all of which were inhibited in the E14-Sox2^TA/WT^ teratomas (Fig. 6c). Analysis of upstream regulators showed that Mknk1, Sox2, Psmb11, and Mecp2 function was inhibited, whereas Trp53 function was activated (Fig. 6d). These results suggest that Sox2 T258 *O-*GlcNAcylation may influence the early cell fate of ESCs upon differentiation in vivo.

## Discussion

*O*-GlcNAcylation plays an important role in early embryonic development^46–48^. For determination of how *O*-GlcNAcylation affects cell fate during the early embryonic developmental stages, it is necessary to investigate the mechanisms by which *O*-GlcNAcylation alters the functions of key factors of embryonic development. Oct4, Sox2, Nanog, Klf4, Klf2, Tbx3, Tfcp2l1, and Esrrb are key factors governing the ground-state ESC transcription circuit^49^, and *O*-GlcNAcylation of Oct4, Sox2, and Esrrb has been reported^3,5,20,21^. *O*-GlcNAcylation at one residue in Oct4 and Esrrb and at two residues in Sox2 has been observed. In mouse ESCs, Oct4 S229 *O*-GlcNAcylation enhanced the transcription of pluripotency-related genes, whereas Esrrb S25 *O*-GlcNAcylation enhanced protein stability, protein–protein interactions, and transcriptional activity^3,5^. In both cases, *O*-GlcNAcylation contributed to enhanced ESC self-renewal. Sox2 S248 and T258 residues are *O*-GlcNAcylated under ESC self-renewal conditions^20,21^. *O*-GlcNAcylation of Sox2 at S248 has been reported to negatively affect ESC self-renewal^21^, which is logically difficult to comprehend given that overall *O*-GlcNAcylation of Sox2 is reduced upon induction of differentiation^21^. Therefore, for further elucidation of the role of Sox2 *O*-GlcNAcylation in ESCs, the role of *O*-GlcNAcylation at each residue of Sox2 was investigated. Because ESC self-renewal is sensitive to the level of Sox2 protein expression^50,51^, experiments comparing WT and mutant Sox2 without considering expression levels may result in erroneous conclusions. Thus, the functions of the Sox2 S248A and T258A mutants were investigated in ESCs in which endogenous Sox2 was removed and exogenous Sox2 was expressed only in the amount originally present. Myers et al. showed that Sox2 S248A can replace WT Sox2 in mouse ESCs^21^. In the present study, the Sox2 T258A mutation, but not the S248A and S248D mutations, reduced the ESC self-renewal potential (Fig. 1b). The Sox2 S248A/T258A double mutant restored the reduced self-renewal potential caused by the T258A mutation (Fig. 1d). These results are logically consistent with previous studies reporting that both Sox2 S248A and S248D increase somatic cell reprogramming efficiency^21^ and that the Sox2 T258A and S259A double mutant decreased reprogramming efficiency, whereas the S248A/T258A/S259A triple mutant did not^3^. Considering the results of this study together with those of previous reports, ESC self-renewal potential may be positively regulated by *O*-GlcNAcylation at the Sox2 T258 residue but negatively regulated by *O*-GlcNAcylation at the S248 residue. Further studies are needed to determine why *O*-GlcNAcylation plays opposing roles depending on the residue type in the Sox2 protein.

In addition, nonphysiological outcomes may result from ESCs expressing Sox2 via exogenous promoters, as Sox2 regulation also varies at the transcriptional level. Therefore, in the present study, to investigate the function of Sox2 T258 *O*-GlcNAcylation under conditions closer to the actual physiological state, we expressed Sox2 T258A by introducing a mutation into an endogenous chromosome using the CRISPR/Cas9 gene-editing system. E14-Sox2^TA/WT^ cells, in which the T258A mutation was introduced into only one allele, exhibited a significant decrease in self-renewal (Fig. 2d, e). No ESCs contained the Sox2 T258A mutation in either allele, suggesting that the reduced self-renewal of E14-Sox2^TA/WT^ is due to haploinsufficiency rather than the dominant-negative role of Sox2 T258A. If Sox2 T258A had a dominant-negative role, Sox2 T258A heterologous and homologous mutations would affect self-renewal to a similar extent, resulting in ESCs containing Sox2 T258A homologous mutations. To introduce mutations using the CRISPR/Cas9 system, we separated ESCs into single cells, with only one cell placed in each well of a culture dish for culturing. Cells harboring the T258A mutation in both alleles might have had insufficient self-renewal potential to undergo amplification from a single cell. The results obtained using the E14-Sox2^TA/WT^ cells clearly showed that inhibition of Sox2 T258 *O*-GlcNAcylation reduced the self-renewal of ESCs.

Sox2 also plays an important role in cell fate determination during the early differentiation of ESCs^15,16^, but the role of *O*-GlcNAcylation in cell fate determination has not been reported previously. Because ESCs expressing Sox2 via exogenous promoters exhibit a significantly different differentiation pattern compared with normal ESCs, the role of Sox2 T258A in the differentiation process could only be accurately investigated using E14-Sox2^TA/WT^ cells. From the examination of teratoma formation in nude mice, the most pronounced differences in the E14-Sox2^TA/WT^-derived teratomas compared with the E14-derived teratomas were a reduction in the number of cells with an ectodermal lineage and increased cartilage formation (Fig. 5). Sox2 has been reported to contribute to ectoderm formation by repressing the expression of mesendodermal genes via physical interactions with their enhancers^13,52^. Additionally, Sox2 has been reported to be a negative regulator of osteoblast maturation in vivo^53^. Considering these findings, the decrease in ectodermal lineage commitment seems to be because Sox2 T258A does not effectively inhibit mesendodermal lineage commitment and osteoblast maturation.

We found that 2TS22C FLAG-Sox2 T258 cells in which Sox2 WT was completely replaced with T258A showed reduced but still maintained self-renewal (Fig. 1). Given that Sox2 is essential for self-renewal, Sox2 T258 O-GlcNAcylation appears to fine-tune the function of Sox2. *O*-GlcNAcylation of proteins usually affects protein stability, localization, or interactions with other partners^2^. Sox2 T258 O-GlcNAcylation did not significantly affect the protein stability or localization of Sox2 (Fig. 2b, c, Supplementary Fig. 2, and Supplementary Fig. 3). Sox2 T258 O-GlcNAcylation does not seem to affect DNA binding, as Sox2 T258 is present in the transactivation domain rather than the DNA binding domain (Fig. 1a), and the expression of most Sox2 direct targets was not altered, as shown by RNA-seq analysis (Fig. 4a). Given that Sox2 often regulates the expression of target genes in conjunction with other cofactors^15^ and the finding that Sox2 S248 O-GlcNAcylation regulates the binding of Sox2 with other cofactors^21^, Sox2 T258 O-GlcNAcylation likely affects the binding of Sox2 to other cofactors. Further studies are needed to determine which cofactors are regulated by Sox2 T258 O-GlcNAcylation and what happens as a result.

**Fig. 7:**
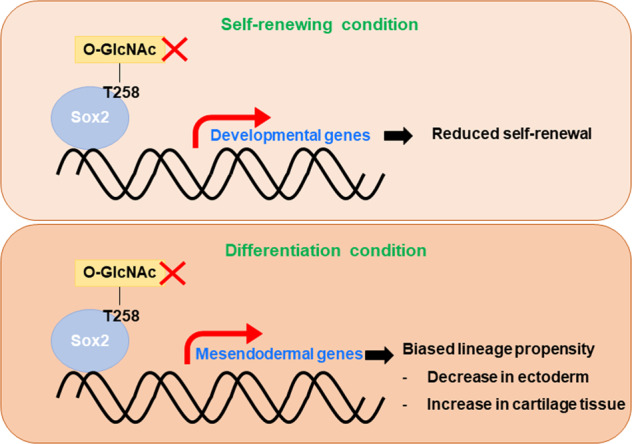
Model describing the role of Sox2 T258 *O*-GlcNAcylation in ESCs. The Sox2 *O*-GlcNAcylation level is high under ESC self-renewal conditions and decreases upon ESC differentiation. When Sox2 T258 *O*-GlcNAcylation is inhibited, the suppression of developmental genes by Sox2 does not occur properly, decreasing ESC self-renewal. In addition, Sox2 T258 *O*-GlcNAcylation is important for the development of ectoderm through the repression of mesendodermal genes. The reduction in Sox2 T258 *O*-GlcNAcylation during early embryonic development decreases the number of cells committed to an ectodermal lineage.

In this study, we revealed that Sox2 T258 *O*-GlcNAcylation positively affects ESC self-renewal and is important for the repression of mesendodermal genes during the early differentiation stage of teratoma formation (Fig. 7). These findings allow us to draw a direct link between Sox2 *O*-GlcNAcylation and early cell fate decisions.

## Supplementary information

Supplementary information

Movie S1

Movie S2
