# Supplementary material for: O-GlcNAcylation of Sox2 at threonine 258 regulates the self-renewal and early cell fate of embryonic stem cells
Source: Exp Mol Med. 2021 Nov 24;53(11):1759–68. doi: 10.1038/s12276-021-00707-7 (PMC8639819; doi:10.1038/s12276-021-00707-7)
Supplement: Supplementary file 1 — Supplementary information [file 12276_2021_707_MOESM1_ESM.pdf]

Supplementary Information

O-GlcNAcylation of Sox2 threonine 258 regulates the self-renewal and early cell fate propensity of embryonic stem cells

Dong Keon Kim[1,*], Jang-Seok Lee[1,2,*], Eun Young Lee[1,3], Hansol Jang[1,4], Suji Han[1], Hee Yeon Kim[1], In-Young Hwang[2,5], Ji-Woong Choi[6], Hyun Mu Shin[6,7], Hye Jin You[1,4], Hong-Duk Youn[2,3], Hyonchol Jang[1,4]

List of Supplementary Information

Supplementary Fig. 1. (Related to Fig. 1b) Similar retroviral titers of Sox2 wild type and mutants.

Supplementary Fig. 2. (Related to Fig. 2b) Cycloheximide chasing analysis showed that Sox2 proteins from E14 and E14-Sox2TA/WT cells have similar protein stability.

Supplementary Fig. 3. (Related to Fig. 2c) Three-dimensional confocal microscopy analysis of Sox2 subcellular localization in 2TS22C cells containing Sox2 wild type and T258A.

Supplementary Fig. 4. (Related to Fig. 5a) Full images of teratomas derived from E14 and E14-$Sox2^{TA/WT}$ cells.

Supplementary Movies 1 and 2. (Related to Fig. 2d) Time-lapse image of E14 and E14-$Sox2^{TA/WT}$ cells under self-renewing condition. Supplementary Movie 1, E14; Supplementary Movie 2, E14-$Sox2^{TA/WT}$

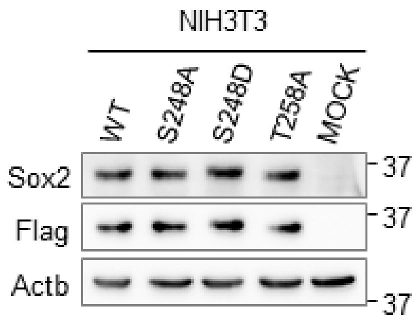

Supplementary Fig. 1. (related to Fig. 1b) **Similar retroviral titers of Sox2 wild type and mutants**

Equal numbers of NIH3T3 cells were infected with the same amounts of retroviruses used to infect 2TS22C cells. Expression level of Sox2 WT and mutants were evaluated by Western blot analysis. Actb was used as a loading control.

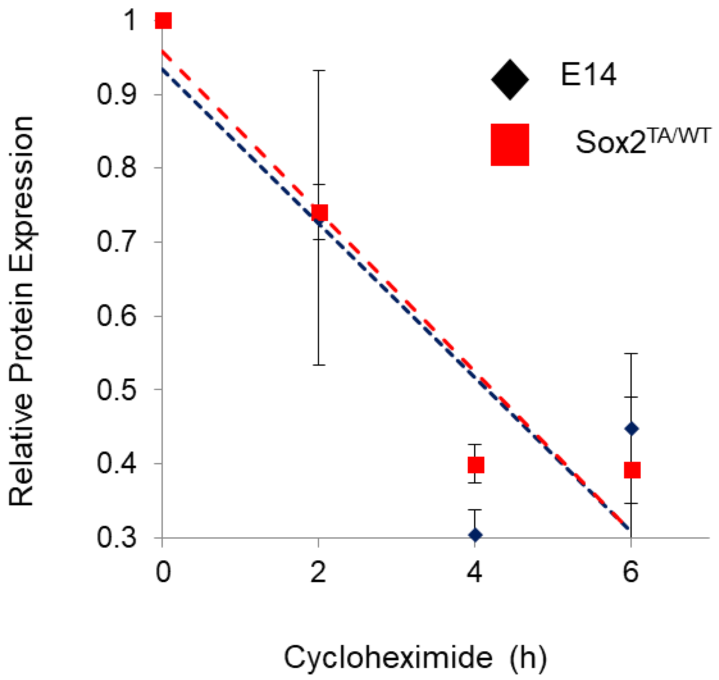

Supplementary Fig. 2. (related to Fig. 2b) **Cycloheximide chasing analysis showed that Sox2 proteins from E14 and E14-Sox2TA/WT cells have similar protein stability.**

Cells were treated with cycloheximide (20 µg/ml) for indicated time periods and the Sox2 protein levels were determined by Western blot. Sox2 protein levels were normalized by Actb protein levels and relative normalized Sox2 protein expression levels relative to untreated controls were indicated.

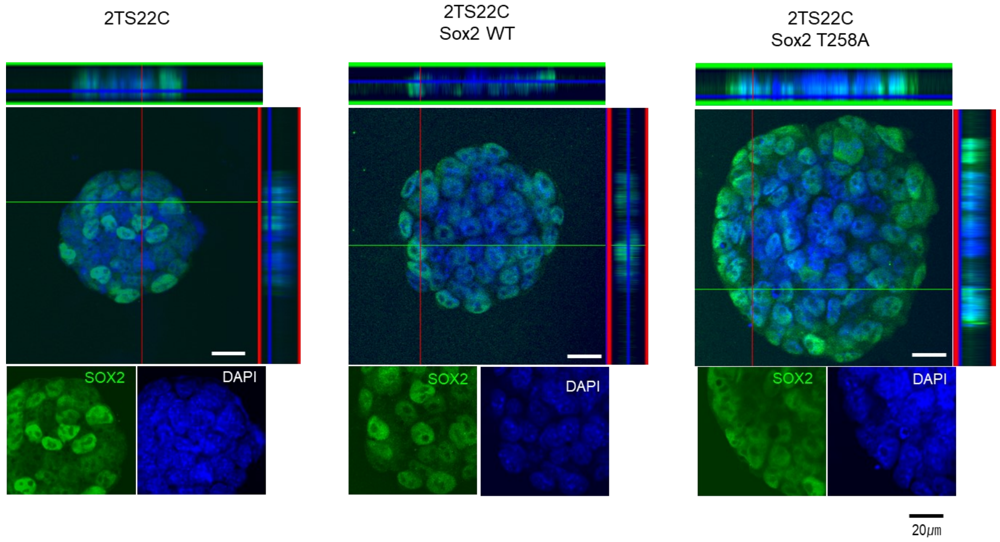

Supplementary Fig. 3. (related to Fig. 2c) **Three-dimensional confocal microscopy analysis of Sox2 subcellular localization in 2TS22C cells containing Sox2 wild type and T258A.**

The subcellular localization of Sox2 wild-type and T258A in 2TS22C cells was analysed by immunofluorescent staining and 3D-confocal microscopy. Representative Z-stack images and images from one focal plane are shown. Scale bar, 20 μm.

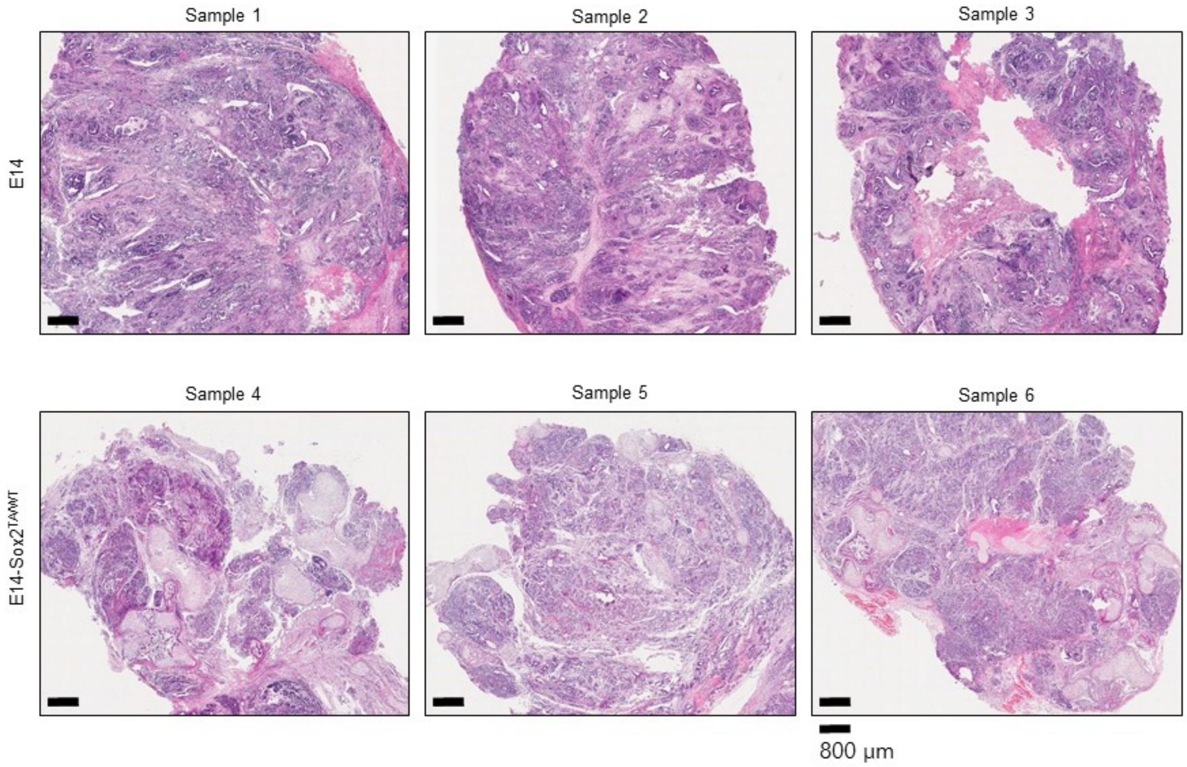

Supplementary Fig. 4. (Related to Fig. 5a) **Full images of teratomas derived from E14 and E14-Sox2$^{TA/WT}$.**

Three nude mice per group were injected subcutaneously with E14 or E14-Sox2$^{TA/WT}$ cells and then sacrificed 14 days later for teratoma analysis. Representative full images of hematoxylin & eosin (H&E) staining for each teratoma are shown. Scale bar, 800 μm.
